# Eating behavior among persons with type 2 diabetes mellitus in North Ethiopia: a cross-sectional study

**DOI:** 10.1186/s12902-021-00750-5

**Published:** 2021-05-17

**Authors:** Hagos Amare Gebreyesus, Girmatsion Fisseha Abreha, Sintayehu Degu Besherae, Merhawit Atsbha Abera, Abraha Hailu Weldegerima, Eshetu Girma Kidane, Afework Mulugeta Bezabih, Tefera Belachew Lemma, Tsinuel Girma Nigatu

**Affiliations:** 1 Department of Nutrition & Dietetics, Jimma University, Jimma, Ethiopia; 2 College of Health Sciences, Mekelle University, Mekelle, Ethiopia; 3 Department of Preventive Medicine, Addis Ababa University, Addis Ababa, Ethiopia; 4 Department of Pediatrics and Child Health, Jimma University, Jimma, Ethiopia; 5 Jimma University Clinical and Nutrition Research Center (JUCAN), Jimma University, Jimma, Ethiopia

**Keywords:** T2DM, Eating behavior, North Ethiopia

## Abstract

**Background:**

Diet is central to the management of type 2 diabetes mellitus (T2DM). Depending on the stage of the disease at which the recommended diet is initiated, optimal adherence can reduce HbA1c by about 1 to 2%. However, evidence on eating behavior is generally scarce including in Ethiopia. The present study aimed to assess the eating behavior of adults with T2DM in North Ethiopia.

**Methods:**

This cross-sectional study was conducted among 421 adults with T2DM from September to November 2019. Socio-demographic variables were collected using structured questionnaires; an asset-based wealth index was used to determine socioeconomic status. Three dimensions of eating behavior were assessed using Likert-type items: food selection, meal planning and calorie recognition. Raw Likert scores in each dimension were transformed to percent scales to maximum (%SM). Participants’ behavior in each dimension was categorized into healthy and unhealthy taking 66.7% SM score as a cutoff. Overall eating behavior was determined by aggregating ranks scored in the three dimensions. Correlates of overall eating behavior were identified using Chi-square test and multinomial logistic regression with statistical significance set at *P*-value < 0.05.

**Result:**

Only 1% of the participants had overall healthy eating behavior. Yet, overall unhealthy eating was apparent in 54.4%. By dimensions, healthy eating behaviors in food selection, meal planning and calorie recognition were seen in 43.5, 7.4 and 2.9% participants, respectively. Factors that were positively associated with having healthy eating behavior in one dimension relative to unhealthy in all were: receiving nutrition education [AOR 1.73; CI 1.09, 2.74], female gender [AOR 1.78; CI 1.03, 3.08] & being in 26–44 age category [AOR 3.7; CI 1.56, 8.85]. But, being in the poor [AOR 0.42; CI 0.16, 1.32] or average [AOR 0.54; CI 0.19, 1.55] socioeconomic strata were negatively associated. However, only receiving nutrition education [AOR 3.65; CI 1.31, 10.18] was significantly associated with having healthy behavior in two eating dimensions over unhealthy in all.

**Conclusion:**

In North Ethiopia, the overall eating behavior of adults with T2DM is extremely poor. Diverse and integrated approaches including nutrition education during consultation should be implemented to address the gap.

**Supplementary Information:**

The online version contains supplementary material available at 10.1186/s12902-021-00750-5.

## Background

Diabetes mellitus is the most challenging public health threat of the twenty-first century [1]. Globally, an estimated 451 million individuals had diabetes in 2017, which is projected to rise to 693 million by 2045 [2]. Type 2 diabetes mellitus (T2DM) accounts for approximately 90% of diabetes cases and drives the pandemic [2].

A major concern in diabetes is the complications that occur due to long-standing hyperglycemia. The complications including cardiovascular diseases, neuropathy, nephropathy and retinopathy lead to disability and mortality [2]. Hence, optimal glycemic control is fundamental to prevent and/or delay these complications. Such improved glycemic control demands to be underpinned by self-management measures [3].

Self-management is the process of actively engaging in self-care activities with the goal of improving one’s behavior and well-being [4]. Diabetes self-management includes regular exercise, taking a recommended diet, proper intake of prescribed medications, and blood glucose monitoring. Dietary self-management promotes healthy eating and assists in achieving healthy weight, blood glucose, lipid, and blood pressure goals more than other aspects of diabetes self-management [5, 6].

Healthy eating is an integral part [7] and a cornerstone of diabetes self-management practices [8]. Taking this into consideration, evidence-based nutrition recommendations have been formulated by various organizations [9–11]. These recommendations promote healthy eating and advice people with diabetes to select healthy foods, arrange their meal plan, and manage their calorie needs. More specifically persons with T2DM are recommended to eat whole grains, beans, fruits, and non-starchy vegetables that supply fiber, important vitamins, minerals, and antioxidants and rarely serve from fats, oils, sweets and alcohol [9–12]. Salt should also be consumed in a limited amount, only about 2300 mg/day [12]. Moreover, patients should prepare a meal plan that portrays eating six times a day comprising 3 meals and 3 snacks and also account for food variety, portion size, and serving time. Additionally, persons with T2DM have to consider their daily calorie consumption in relation to body weight, type of activity, and associated illness if any [10, 11].

But, pieces of evidence from the US [13], Europe [14, 15], Asia [16, 17] and, Africa [18] show that most individuals with T2DM have difficulty of long-term adherence with dietary recommendations. In Ethiopia, though, the evidence is scanty the available few studies show poor dietary practice among individuals with T2DM that ranges from 48.6 to 74.3% [19–23]. However, data on eating behavior of adults with T2DM from Tigray region is lacking. This study aimed to fill the evidence gap on eating behavior of persons with T2DM that could enhance evidence-based policymaking and decision for diabetes care programs in Ethiopia.

## Methods

### Study setting

The study was conducted in Adigrat and Mekelle Hospitals, Tigray, North Ethiopia. Both Hospitals have the status of a general Hospital and provide diabetes care services in their clinics. Mekelle General Hospital (MGH) is located in Mekelle city, which is situated about 780 km North of Addis Ababa. The total population of Mekelle was 215,914 according to census 2007 [24]. The MGH provides a long term multifaceted health service including diabetes care. About 684 persons with diabetes were under follow-up during the study period. Adigrat General Hospital (AGH) is located in Adigrat, a populous city (57,588 again according to census 2007) in Tigray next to Mekelle. It is located about 900 km North of Addis Ababa and 35 km south of the border of Eritrea. The Hospital offers a range of health care services for the population in the eastern zone of Tigray including occupants of Adigrat. There were about 527 persons with diabetes under follow-up up during the study period.

### Study design and study period

A cross-sectional quantitative study was conducted from September to November, 2019.

### Study participants

The participants of this study were adults with T2DM enrolled in the diabetic follow-up clinics of AGH and MGH, North Ethiopia. Having T2DM, being 18 years and above, and visiting the clinics in the study period were the inclusion criteria. Patients who were pregnant, breast feeding, and/or having documented cognitive impairment were excluded.

### Sample size and sampling technique

The sample size was calculated using a 51.4% prevalence of poor dietary practice among persons with T2DM in Addis Ababa, Ethiopia [19]. We used single population proportion formula to determine sample size of 422 considering reliability coefficient Z = 1.96 at 95% confidence interval, margin of error (d) = 0.05 and 10% non-response rate. The sample was proportionately allocated to the two hospitals based on the number of patients under follow-up. Accordingly, 237 and 184 participants were selected from MGH and AGH, respectively. We did systematic sampling making use of the appointment list obtained from the logbook as a frame.

### Instruments and measures

We used three types of data collection tools. Demographic variables were collected using a structured questionnaire prepared by the study team. Household socio-economic variables were collected using a tool validated for Demographic Health Survey (DHS) [25]. For data on eating behavior, we adapted a tool from a similar Bhutanese study [26]. All questionnaires were prepared in English and translated into the local language, Tigrigna.

The Bhutanese tool had a total of 19 Likert-type items. Eight items were used to measure food selection dimension of the eating behavior. Seven items were utilized to measure meal planning dimension and the remaining four items for calorie needs recognition dimension. The instrument was pretested and checked for internal consistency for each dimension. Accordingly, all the items for meal planning and calorie recognition dimensions were found to qualify internal consistencies with Cronbach’s Alpha coefficient (α) 0.79 & 0.84, respectively. The pretest showed that one item each from meal planning and calorie recognition dimension was not understandable by the participants. Removal of these items thus slightly reduced the α for meal planning and calorie needs recognition dimensions to 0.74 & 0.71, respectively. For the food selection dimension, five of the eight items were retained to achieve an acceptable level of internal consistency at α of 0.58. Based on the psychometric evaluation, 14 of the 19 items were finally valid for assessing the overall eating behavior of the participants.

Likert-type items in each dimension had four response anchors: strongly disagree, disagree, agree and strongly agree. They were given equal weight and assigned with equidistant points that range from 1 for strongly disagree to 4 for strongly agree. The Likert-type item scores in each eating behavior dimensions were computed into Likert-scale scores. In order to ease comparison with studies that may utilize a different number of items or response anchors, the raw scores were transformed to percentage of scale to maximum score (%SM) [27]. Since the value for the minimum possible score in our Likert-type item was one, the % SM range was 0 up to 100.

Participants’ behavior in each dimension was categorized in to ‘healthy (% SM ≥ 66.7)’ and ‘unhealthy (% SM < 66.7)’. The ground for the % SM cutoff value was the participants’ reaction to the Likert-type items (questions) under each dimension. Subjects who expressed their disagreement (disagree or strongly disagree) to the characteristic represented in a particular Likert-type item have a % SM score of less than 66.7. While participants who uttered their agreement (agree or strongly agree) to the trait have a % SM score of greater than or equal to 66.7.

The overall eating behavior of the participants was evaluated by considering their aggregate ranks in food selection, meal planning, and calorie recognition dimensions. A participant was labeled as having overall healthy eating behavior if he/she had achieved a % SM score of 66.7 or greater in all of the three dimensions. In the same token, a participant was labeled as having overall unhealthy eating behavior if he/she had achieved a % SM score of below 66.7 in both dimensions. Participants who didn’t satisfy the scores for the stated cutoff in all dimensions but have an acceptable score/s in one or two were reported as healthy in one or healthy in two dimensions, respectively. Consequently, overall eating behavior has been grouped into four.

### Statistical analysis

Data were checked for completeness and entered in to Epidata 3.1 (Xunta de Galicia, Spain & PAHO, USA) and analyzed using SPSS for windows version 23 (IBM Corp, New York). Wealth index score was generated using Principal Components Analysis (PCA), for urban and rural participants separately. In each case, the first PCA factor that explained most of the variation was used to group study subjects into three wealth quintiles. Finally, the quintiles from urban and rural participants were merged by cases for final inclusion of socioeconomic status as an explanatory variable in regression model.

Normality was checked for all metric variables. Means and standard deviations were reported when pertinent. Categorical variables were summarized using frequencies and percentages. Pearson product-moment correlation coefficient was computed to assess the correlation among the eating behavior dimensions. To investigate the association of socio-demographic and nutrition information features with overall eating behavior as an outcome; the chi-square test and multinomial logistic regression analysis were carried out. The fourth group in the overall eating behavior i.e. healthy in three dimensions had insufficient count and hence merged with the third one. Healthy behavior in two dimensions was considered as a reference against which the other two responses compared.

A chi-square test was used to assess the simple relationship between each presumed factor and the overall eating behavior of participants. Multinomial logistic regression analysis was performed to identify significant contributors to the overall eating behavior after adjusting for potential confounders. The decision to fit explanatory variables into the final model was made based on results from bivariate analysis and logical reasoning. The presumed factors were tested for multicollinearity as well and none of them had shown a considerable correlation. The level of statistical significance was set at 5% (*p* < 0.05).

### Ethical considerations

The research proposal was approved by the Institutional Review Board of the Institute of Health, Jimma University. Permission letters were secured from Tigray Regional Health Bureau and participating institutions (Adigrat and Mekelle General Hospitals). Individuals who fulfilled the criteria were provided with information about the study ahead of enrolment to help them understand and make decisions. A personal signature or thumb print was obtained on the consent form to ensure their informed verdict. Data confidentially was kept throughout the course of the study and afterwards.

## Results

### Characteristics of the study participants

We studied 421 individuals with T2DM, of which 53.7% (226) were female, had mean age (±SD) of 58.2 (±11) years. About 97.4% were Tigreans, and 91.9% were followers of Orthodox Christianity (Table 1). Their follow-up period at diabetic clinic ranged from 1 to 30 years with median (IQR) of 5 (6) years.

**Table 1:** Demographic and nutrition information related features of type 2 diabetes patients, (*n* = 421)

Variable	Frequency (%)
**Age in years**	
26–44	50 (11.9)
45–64	231 (54.9)
65+	140 (33.3)
**Residence**	
Urban	351 (83.4)
Rural	70 (16.6)
**Ethnicity**	
Tigrean	410 (97.4)
Amhara	6 (1.4)
Oromo	3 (0.7)
Afar	2 (0.5)
**Religion**	
Orthodox Christian	387 (91.9)
Muslim	21 (5)
Catholic	9 (2.1)
Protestant	4 (1)
**Marital status**	
Single	53 (12.6)
Married	225 (53.4)
Widowed	52 (12.4)
Divorced	91 (21.6)
**Wealth quintile**	
Poor	141 (33.5)
Average	140 (33.3)
Rich	140 (33.3)
**Received nutrition education**	
Yes	280 (66.6)
No	143 (34)
**Nutrition education provider**	
Medical doctor	159 (56.8)
Nurse	78 (27.9)
Medical doctor & nurse	39 (13.9)
Nutritionist	4 (1.5)
**Media of nutrition education**	
Oral	200/280 (71.4)
Written	12/280 (4.3)
Both oral and written	68/280 (24.3)

Regarding receiving nutrition information, 66.6% (280) participants reported to have received at least once. About 56.8% (159) obtained it from their doctors during heath examination and 1.5% (4) from a nutrition expert. The information was received orally or through written nutrition education materials, 71.4% (200) and 4.3% (12) respectively (Table 1).

### Eating behavior

**Fig. 1:**
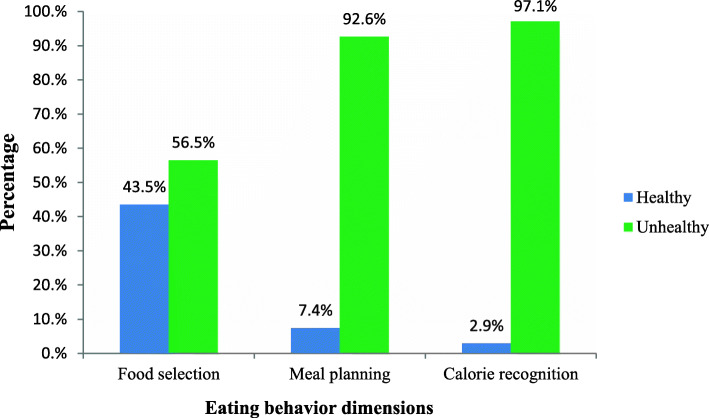
Eating behavior of adults with type 2 diabetes by eating behavior dimensions (*n* = 421). Note: Healthy = Participants whose % SM score is > = 66.7 (their responses rated agree, strongly agree or a mix of the two) in a given dimension of eating behavior, Unhealthy = Participants whose % SM score is < 66.7 (their responses rated strongly disagree, disagree or a mix of the two) in a given dimension of eating behavior

**Fig. 2:**
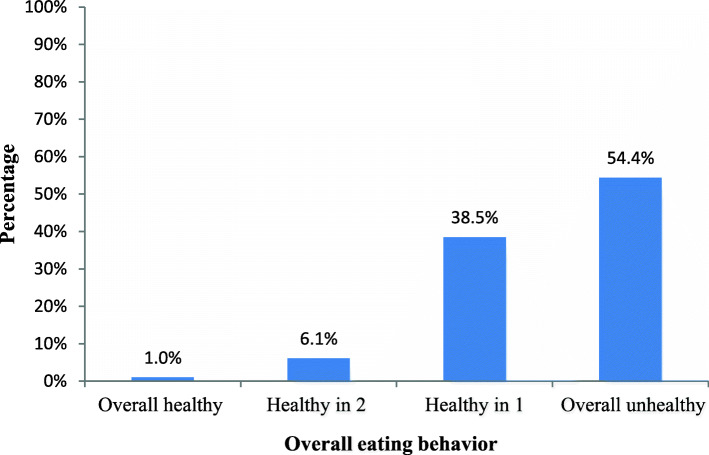
Overall eating behaviors of adults with type 2 diabetes (*n* = 421). Note: Overall healthy = having a %SM score of > = 66.7 in 3 of the dimensions of eating behavior, Healthy in 2 = having a %SM score of > = 66.7 in two dimensions, Healthy in 1 = having a %SM score of > = 66.7 in one dimension & overall unhealthy = having a %SM score of < 66.7 in all three dimensions

All in all the persons with T2DM assessed in this study had unhealthy eating behavior. As indicated in Table 2, the participants’ respective mean and SD of %SM on food selection, meal planning, and calorie recognition dimensions were 59.8 (17.9), 29.2 (20.9), and 24.4 (17.8). About 43.5% (183) of participants had healthy eating behavior on food selection. Nonetheless, only 7.4% (31) and 2.9% (12) of them had healthy eating behavior in meal planning and calorie recognition dimensions, respectively (Fig. 1). Correspondingly, the overall eating behavior was also unsatisfactory. Only 1.0% (4) of the participants had healthy behavior in all the three eating dimensions while 54.4% (229) had unhealthy in all the three (Fig. 2). All the dimensions had positive correlation with stronger relationship observed between meal planning and calorie needs recognition (*r* = 0.63, *p* < 0.001). Though weaker, food selection and meal panning (*r* = 0.38, *p* < 0.001) and food selection and calorie needs recognition (*r* = 0.22, *p* < 0.001) were also correlated.

**Table 2:** Scores of eating behavior dimensions among adults with type 2 diabetes (*n* = 421)

Eating behavior domain	Possible score^b^	Actual score^c^	Mean^a^	%SM^d^
Food selection	5–20	7–20	14.0 (2.7)	59.8 (17.9)
Meal planning	6–24	6–21	11.2 (3.7)	29.2 (20.9)
Calorie recognition	3–12	3–9	5.2 (1.6)	24.4 (17.8)

^a^Data in cell are mean and SD

^b^Range of Likert-scores possibly attained in the given domain

^c^Range of Likert-scores practically attained in the given domain

^d^standardized score as the percentage of scale to maximum possible score, and it lies between 0 and 100

Among, discrete items in the food selection dimension, eating fruits and vegetables daily was the least adhered to behavior while avoiding or taking alcohol in moderation was the most adhered, 9.5% (40) vs. 63.2% (266), respectively. From the meal planning dimension, maintaining serving time was better complied while arranging daily meal using plate method is least exercised, 16.6% (70) vs.1.9% (8), respectively. As shown in Table 3, the participants had negligible practice of weighing food and maintaining calorie proportions taken from calorie-genic nutrients in a daily meal, 0.7% (3) vs. 1.2% (5), respectively.

**Table 3:** Eating behavior of adults with type 2 diabetes by domain and item (*n* = 421)

Dimension with items	Agreement in Likert-scale^a^			
	1	2	3	4
**Food selection**				
1. You choose foods that contain low to medium glycemic index in your diet	19.7	21.6	40.4	18.3^b^
2. You or the person who cooks for you rarely uses saturated fats for cooking	34.4	15.9	16.9	32.8
3. You eat fruits and vegetables every day	22.3	44.9	23.3	9.5
4. You avoid salty diet	5.5	10.2	29.7	54.6
5. You avoid or take alcohol in moderation	8.6	7.8	20.4	63.2
**Meal planning**				
6. You understand and able to arrange your right meal plan	59.6	14	18.1	8.3
7. You understand and able to use plate methods in arranging your meal plan	65.1	20.4	12.6	1.9
8. You understand and able to use food exchange list in arranging your meal	56.1	19.2	19.2	5.5
9. You eat a variety of foods in every meal daily	60.1	20.9	15.9	3.1
10. You eat 3 meals and 3 snacks a day	30.9	41.3	22.3	5.5
11. You eat meal in the same time every day	16.4	22.1	44.9	16.6
**Recognizing amount of calorie needs**				
12. You know and maintain the calorie proportions you should take in each meal	70.5	21.6	6.7	1.2
13. You weight and measure calorie of foods in each meal	69.4	23.8	6.2	0.7
14. You consume same amount of food every day	21.1	25.2	42.2	11.2

^a^**1 - Strongly disagree, 2 - Disagree, 3 – Agree and 4 - Strongly Agree**

^b^ - %

### Factors associated with eating behavior

Of the eight covariates in the bivariate model, age category (*p*-value = 0.033), economic status (*p*-value = 0.032) and nutrition education (*p*-value = 0.025) were positively and significantly associated with overall eating behavior. However, one more factor i.e. sex attained significance level in the adjusted model that simultaneously handles the logits for healthy behavior in one eating dimension versus unhealthy in all the three and healthy in two dimensions versus unhealthy in all (Table 4).

**Table 4:** Factors associated with eating behavior of adults with type 2 diabetes (*n* = 421)

Characteristic	Healthy in one dimension	Healthy in two dimensions
	AOR (95% CI)	AOR (95% CI)
Age		
26–44 years	3.72 (1.56, 8.85) *	0.7 (0.20, 3.15)
45–64	1.04 (0.64, 1.67)	0.65 (0.23, 1.84)
65+	Ref.	
Sex		
Male	Ref.	
Female	1.78 (1.03, 3.08)*	1.57 (0.59, 4.12)
Religion		
Orthodox	Ref.	
Others	0.54 (0.25, 1.18)	1.59 (.17, 4.32)
Residence		
Urban	Ref.	
Rural	1.03 (0.56, 1.89)	2.64 (0.55, 12.75)
Marital status		
Single	Ref.	
Married	0.98 (0.50, 1.89)	0.49 (0.13, 1.83)
Divorced	1.15 (0.48, 2.78)	1.38 (.19, 9.87)
widowed	1.41 (0.62, 3.16)	1.58 (0.26, 9.64)
Educational status		
Illiterate	2.00 (1.00, 3.98)	2.92 (0.85, 10.01)
Read & write	1.41 (0.68, 2.92)	1.87 (0.54, 6.49)
Primary level	1.71 (0.79, 3.70)	1.93 (0.56, 6.68)
Secondary level	2.39 (0.89, 6.4)	2.47 (0.47, 13.04)
College & above	Ref.	
Economic status		
Poor	0.53 (0.31, 0.91)*	0.42 (0.16,1.13)
Average	0.44 (0.26, 0.75)*	0.54 (0.19, 1.55)
Rich	Ref.	
Nutrition education		
Received	1.73 (1.09, 2.74)*	3.65 (1.31, 10.18)*
Not received	Ref.	

Reference-unhealthy in all dimensions

Model fitting: X^2^(df) = 53.2 (30), *P* = 0.006, Goodness- of -fit, *P* = 0.391; Pseudo *R*^*2*^ = 14.3%; AOR = Adjusted Odds ratio; *significant variable (*P*. value < 0.05). CI – Confidence interval; Overall unhealthy = having unhealthy eating behavior in all the three dimensions, Healthy in 1 = having healthy behavior in one dimension out of the three, Healthy in 2 = having healthy behavior in two dimensions out of the three

Accordingly, the factors that were associated with increasing odds of having healthy behavior in one eating dimension relative to unhealthy in all were 26–44 age category [AOR 3.7; CI 1.56, 8.85], female gender [AOR 1.78; CI 1.03, 3.08] and nutrition education [AOR 1.73; CI 1.09, 2.74]. In contrast, being in the poor [AOR 0.53; CI 0.31, 0.91] and average [AOR 0.44; CI 0.26, 0.75] quintiles of the socioeconomic strata were associated with decreasing odds of having healthy in one eating dimension relative to unhealthy in all.

On the other hand nutrition education was the only factor associated with increasing the likelihood of having healthy behavior in two eating dimensions relative to unhealthy in all at a significant odds ratio [AOR 3.65; CI 1.31, 10.18]. Residence, religion, marital status, and educational status were variables that didn’t show a relationship of statistical relevance in both models. The final model was fitted well at X^2^ (df) = 53.2 (30), *P* = 0.006; Pseudo *R*^*2*^ = 14.3% as compared to the null model.

## Discussion

A healthy diet is central to the management of T2DM. In this study we assessed eating behavior of persons with T2DM in North Ethiopia based on three dimensions. Our results indicated that the overall eating behavior was unhealthy. Among the behavior dimensions, food selection was better practiced. However, the performances in meal planning and calorie needs recognition were markedly low. Age category, sex, socioeconomic status, and nutrition education appeared to be factors that were associated with the overall eating behavior.

Compliance to a healthy diet is extremely important to meet blood glucose targets and prevent complications associated with it. Accordingly, imparting nutrition education soon after diagnosis is considered as the foremost duty of health care providers as it has an auspicious effect in motivating healthy eating [9, 28]. In this study, despite self-reported access to nutrition education by two-thirds of the participants, only 1% had an overall healthy eating behavior. This has enormous implication on health status of the individuals. As a rule, successful behavior change requires motivation, ability to perform the new behavior, and trigger the desired reform and sustain it for long [29, 30]. This undoubtedly necessitates periodic reinforcement from an expert side. However, none of the hospitals included in our study had nutrition expert as a teammate of the diabetes care. Hence, inadequate quality and duration of counseling on healthy eating could be one reason for the lower compliance despite relatively fair exposure to nutrition education [31].

The extremely low compliance to healthy eating behavior in this study is matching with an output from Nepalese study where none of their participants had healthy eating behavior [16]. In addition, it is also in agreement with a report from Saudi where only 6% of persons with T2DM tried to follow a dietary guide [17]. However, it is lower than the findings from Pakistan [32], Yemen [33], and way behind from results of Ethiopian studies in other settings [19–23]. Apart from differences in socio-demographic factors, methodological variations could explain the observed discrepancy. All, except one [21], of the Ethiopian studies cited here used tools with yes/no binary response items unlike the four Likert-type responses in our setting. The dichotomous response restricts the level of agreement respondents could demonstrate about their actual behavior and may create biased scores [34]. In addition, in these studies assessment was judged mainly from food selection behavior. However, the current result was an aggregate of three dimensions. This could possibly explain the lower overall eating behavior in the present study as the meal planning and recognizing calorie needs dimensions were demonstrated to be most difficult to comply.

Our findings are not even concurrent with findings from Indonesian and Bhutanese; that used study tools similar to ours. The overall dietary behaviors of both Bhutanese and Indonesian persons with T2DM were better compared to this study. Despite utilizing comparable tools, difference in the method of classification could be a reason for the inconsistency. In the present study, participants were labeled as having overall healthy eating behavior if they satisfy the scores for it in all the three dimensions. However, in the other two studies, item scores from all dimensions were summated. A raw mean value of the total score was calculated. Finally, the overall eating behavior of the participants was judged based on the centiles of the highest possible score where the mean value falls in. However, in such cases, the mean score will be skewed toward the direction of a dimension with highest number of items. The fact that the dimensions are mutually exclusive but complimentary in function, better performance in one dimension shouldn’t mask the low performance in the other or vice versa. Hence, precludes the summated mean score to represent the overall behavior.

Considering individual dimensions, the eating behavior of persons with T2DM from the Bhutanese study was again better. The authors indicated that the mean eating behavior scores of their participants was moderate across all dimensions [26]. In contrast, the % mean scores for selecting healthy foods (59.83 +/− 17.94), meal planning (29.22 +/− 20.94), and recognizing calorie needs (24.39 +/− 17.83) obtained in the present study were below optimal. Though, not consistent across the dimensions like in Bhutan and this study, the discrete eating behavior dimension scores of participants in the Indonesian study were also higher than the current report [35]. Participants had high score in arranging meal plan. But moderate score in recognizing the amount of calorie needs and selecting a healthy food dimensions. The observed difference in the level of adherence to the behavior dimensions could be due to variation in health literacy among the participants in the different studies. Moreover, remarkably low scores in the meal planning and calorie recognition dimensions of this study might imply the nutrition education focuses merely on instructing how to select their food.

Majority of the studied persons with T2DM were unable to follow the recommended eating behavior implies that some factors do exist. One of the most important factors identified in this study was nutrition education. Persons with T2DM who received nutrition education were nearly two times more likely to have healthy behavior in one eating dimension relative to unhealthy in all the three dimensions compared with their counterparts who didn’t get nutrition education. Similarly, the likelihood of having healthy behavior in two eating dimensions relative to unhealthy in all was almost quadrupled in those who received nutrition education. This is consistent with the findings of Worku et al. that showed persons with T2DM who didn’t get nutrition education were almost five times more likely to have poor dietary practice than their counterparts [19]. Likewise, this finding is in line with additional reports from Ethiopia [20–22], Nepal [16], and Indonesia [35]. However; the findings from experimental studies are inconclusive [36–38] and thus further investigation is warranted.

Sex was another factor found to have significant association with overall eating behavior. Our results showed that women with T2DM were 1.8 times more likely to have healthy eating in one dimension relative to unhealthy in all, than men. Additionally, women had 1.6 times more odds of having healthy eating in two dimensions than men though this was not statistically significant. Previous studies have shown that women have better dietary practices than men [32, 39, 40]. A likely explanation for a higher level of unhealthy eating behavior among men could be related to their working environment. As in most place, men in Ethiopia are more likely to work outside of their homes and hence have higher chance to eat out of home which in turn constraints their choice of healthy diets.

The study also demonstrated a significant association of age with overall eating behavior. Participants in the lower age group were about four times more likely to have healthy behavior in one eating dimension relative to unhealthy in all than those in the old age. Although the observed association was not consistent across the categories of the outcome variable; it is in keeping with other similar studies. Increasing age was negatively associated with attainment of proper eating behavior in studies carried out in Nepal [16], Iran [41], and Canada [42]. In contrast, Ethiopian [20], Bahraini [43] and Spanish [44] authors demonstrated a positive association of increasing age with healthy eating behavior. Like the findings, the justifications around here are inconclusive. Some scholars believe that it is easier to eat healthily at old age as there is wider chance to cook and eat at home at this age. While others argue it is less likely due to memory lapse to remember what they are educated. As our data is insufficient to explain the observed inconsistency we recommend further study as a tie-breaker for the two schools of thought.

Income affects many aspects of eating behavior including food purchasing power. Persons with higher income are more likely to have better access to healthy food and thus have better chance for healthy eating [45]. Our findings reinforce this relationship: persons with T2DM who were from poor and average socioeconomic stratum had lesser odds of having healthy eating compared with the better off. Similar relationships were found by independent studies from Buhtan and Indonesia [26, 35]. Most participants in our study were observed to have the notion that all healthy foods are costly. Nutrition education services and policies should address issues of cost and accessibility of local diet for healthy eating.

Lastly, in general people with higher educational attainment are considered to have better comprehension of nutritional information and healthy eating behavior [46]. Interestingly, we did not find an association between educational status and eating behavior. This is also in contrast with findings of other studies from Ethiopia [47], Iran [41], and Bahrain [43] that revealed better dietary practice among participants with higher educational status. One possibility for the absence of difference in eating behavior by educational status in this study could be lack of accessible dietary guidelines and nutrition behavior change communication outlets even for the educated people to get information.

### Strengths and limitations

A major strength of this study is that it has assessed the eating behavior in three dimensions unlike previous studies that emphasized on food selection and/or rarely meal planning. This study also used Likert-type items rather than the binary response unlike previous studies from Ethiopia on the same area. Moreover, reporting results in standardized scores makes it remarkably adaptable for comparison with other studies. This study, however, has some limitations. The eating behavior of the participants was self-report data collected through an interviewer-administered questionnaire which can suffer from social desirability bias. Besides, perception which is assumed to have a noteworthy share in determining one’s eating behavior was not addressed in this study.

## Conclusion

In general, nearly all persons with T2DM in this study had unhealthy eating behavior. Food selection was better complied whereas meal planning and managing daily calorie needs were seriously breached. The most important factor associated with unhealthy eating behavior was the lack of access to nutrition education services. As diet is a mainstay of diabetes self-management, such divergence from healthy eating is detrimental to the health of the participants. Hence, it is crucial for the health system of Ethiopia, to ensure persons with T2DM establish and sustain healthy eating behavior through designing and implementing thoughtful strategies that help render quality nutrition education service. Moreover; qualitative exploration of the perception that persons with T2DM have towards healthy eating is highly commendable.

## Supplementary Information

**Additional file 1.** Demographic background and nutrition information of participants.

**Additional file 2.** Eating behavior questionnaire (food selection, meal planning and calorie needs recognition).

## Data Availability

The datasets utilized for the current study are available from the corresponding author on reasonable request.

## Abbreviations

AGH: Adigrat General Hospital
AOR: Adjusted odds ratio
DHS: Demographic Health Survey
HbA1c: Hemoglobin A1c
IQR: Interquartile range
MGH: Mekelle General Hospital
T2DM: Type 2 diabetes mellitus
SD: Standard deviation
%SM: Percentage of scale to maximum
US: United States

## References

[1] Zimmet PZ (2017). Diabetes and its drivers: the largest epidemic in human history?. Clin Diabetes Endocrinol.

[2] International Diabetes Federation (2017). Prevalence and projections. IDF Diabetes Atlas.

[3] Inzucchi SE, Bergenstal RM, Buse JB, Diamant M, Ferrannini E, Nauck M, Peters AL, Tsapas A, Wender R, Matthews DR, American Diabetes Association (ADA), European Association for the Study of Diabetes (EASD) (2012). Management of hyperglycemia in type 2 diabetes: a patient-centered approach: position statement of the American Diabetes Association (ADA) and the European Association for the Study of Diabetes (EASD). Diabetes Care.

[4] Powers MA, Bardsley J, Cypress M, Duker P, Funnell MM, Fischl AH (2016). Diabetes self-management education and support in type 2 diabetes: a joint position statement of the American Diabetes Association, the American Association of Diabetes Educators, and the Academy of Nutrition and Dietetics. Clin Diabetes.

[5] Derosa G, Limas CP, Macías PC, Estrella A, Maffioli P. Dietary and nutraceutical approach to type 2 diabetes. Arch Med Sci. 2014;10(2):336–44. 10.5114/aoms.2014.42587 PMC4042055 24904670

[6] Franz M, Boucher JL, Evert AB. Evidence-based diabetes nutrition therapy recommendations are effective: the key is individualization. Diabetes, Metabolic Syndrome and Obesity: Targets Therapy. 2014;7:65–72. 10.2147/DMSO.S45140. 10.2147/DMSO.S45140 PMC3938438 24591844

[7] Yannakoulia M (2006). Eating behavior among type 2 diabetic patients: a poorly recognized aspect in a poorly controlled disease. Rev Diabet Stud.

[8] Ekore RI, Ajayi IO, Ekore JO. Dietary management of diabetes: a practical approach for primary care physicians in Nigeria. Diabetes. 2008;16:13–14.

[9] American Diabetes Association (2008). Nutrition recommendations and interventions for diabetes: a position statement of the American Diabetes Association. Diabetes Care.

[10] The Diabetes and Nutrition Study Group (DNSG) of the European Association for the Study of Diabetes, (EASD) (2000). Recommendations for the nutritional management of patients with diabetes mellitus. Eur J Clin Nutr.

[11] Dworatzek PD, Arcudi K, Gougeon R, Husein N, Sievenpiper JL, Williams SL (2013). Nutrition therapy. Can J Diabetes.

[12] Evert AB, Boucher JL, Cypress M, Dunbar SA, Franz MJ, Mayer-Davis EJ, Neumiller JJ, Nwankwo R, Verdi CL, Urbanski P, Yancy WS Jr, American Diabetes Association (2013). Nutrition therapy recommendations for the management of adults with diabetes. Diabetes Care.

[13] Albright TL, Parchman M, Burge SK. Predictors of self-care behavior in adults with type 2 diabetes: an RRNeST study. Fam Med. 2001;33(5):354–60. 11355645

[14] Rivellese AA, Boemi M, Cavalot F, Costagliola L, De Feo P, On behalf of The Mind.it Study Group (FoRiSID) (2008). Dietary habits in type II diabetes mellitus: how is adherence to dietary recommendations?. Eur J Clin Nutr.

[15] Oftedal B, Bru E, Karlsen B (2011). Motivation for diet and exercise management among adults with type 2 diabetes: motivation for diet and exercise management. Scand J Caring Sci.

[16] Parajuli J, Saleh F, Thapa N, Ali L (2014). Factors associated with nonadherence to diet and physical activity among nepalese type 2 diabetes patients; a cross sectional study. BMC Res Notes.

[17] Alrasheedi AA. Evaluation of dietary habits effect among Saudi patients with type II diabetes mellitus. CRDOJ. 2018;6(4) [cited 2020 Mar 12]. Available from: https://juniperpublishers.com/crdoj/CRDOJ.MS.ID.555695.php.

[18] Zimmermann M, Bunn C, Namadingo H, Gray CM, Lwanda J (2018). Experiences of type 2 diabetes in sub-Saharan Africa: a scoping review. Glob Health Res Policy.

[19] Worku A, Mekonnen Abebe S, Wassie MM (2015). Dietary practice and associated factors among type 2 diabetic patients: a cross sectional hospital based study, Addis Ababa, Ethiopia. SpringerPlus.

[20] Demilew YM, Alem AT, Emiru AA (2018). Dietary practice and associated factors among type 2 diabetic patients in Felege Hiwot Regional Referral Hospital, Bahir Dar, Ethiopia. BMC Res Notes.

[21] Ayele AA, Emiru YK, Tiruneh SA, Ayele BA, Gebremariam AD, Tegegn HG (2018). Level of adherence to dietary recommendations and barriers among type 2 diabetic patients: a cross-sectional study in an Ethiopian hospital. Clin Diabetes Endocrinol.

[22] Mohammed MA, Sharew NT. Adherence to dietary recommendation and associated factors among diabetic patients in Ethiopian teaching hospitals. Pan Afr Med J. 2019;33 [cited 2020 Mar 12]. Available from: http://www.panafrican-med-journal.com/content/article/33/260/full/. 10.11604/pamj.2019.33.260.14463 PMC6814932 31692826

[23] Halima MI, Fisseha Z, Tarkie AW, Abere WA. Knowledge, Practice, and its associated factors of type 2 diabetic patients towards dietary therapy at University of Gondar Specialized Hospital, Northwest, Ethiopia 2017. J Diabetes Clin Prac. 2019;3(1):1–7.

[24] CSA (2007). Population and housing census.

[25] Vyas S, Kumaranayake L (2006). Constructing socio-economic status indices: how to use principal components analysis. Health Policy Plan.

[26] Om P, Deenan A, Pathumarak N (2013). Factors influencing eating behavior of people with type 2 diabetes in Bhutan. J Sci.

[27] Cummins, R.A. On the trail of the gold standard for subjective well-being. Soc Indic Res. 1995;35:179–200. 10.1007/BF01079026.

[28] Madhu S (2015). World diabetes day 2015: healthy living & diabetes. Indian J Med Res.

[29] Ory M. The science of sustaining health behavior change: the health maintenance consortium. ajhb. 2010;34(6) [cited 2020 Mar 12]. Available from: http://www.ingentaconnect.com/content/png/ajhb/2010/00000034/00000006/art00002. 10.5993/ajhb.34.6.2 PMC3753403 20604691

[30] Fogg B (2009). A behavior model for persuasive design. Proceedings of the 4th International Conference on Persuasive Technology - Persuasive ‘09.

[31] Wilson C, Brown T, Acton K, Gilliland S (2003). Effects of clinical nutrition education and educator discipline on glycemic control outcomes in the Indian Health Service. Diabetes Care.

[32] ALhariri A, Daud F, Almaiman A, Saghir SAM. Factors associated with adherence to diet and exercise among type 2 diabetes patients in Hodeidah city, Yemen. Diabetes Manag. 2017;7(3):264–71.

[33] Bano A, Afzal M, Sarwar H, Waqas A, Kousar S, Gulzar S (2017). Dietary knowledge, attitude and practices of diabetes patients at services hospital Lahore. Int J Appl Sci Biotechnol.

[34] Walters SJ (2009). Quality of life outcomes in clinical trials and health-care evaluation: a practical guide to analysis and interpretation.

[35] Primanda CK, Thaniwattananon P. Thaniwattananon. Dietary behaviors among patients with type 2 diabetes mellitus in Yogyakarta, Indonesia. Nurse Media J Nurs. 2011;1(2):211–23. 10.14710/nmjn.v1i2.975.

[36] Wang H, Song Z, Ba Y, Zhu L, Wen Y (2014). Nutritional and eating education improves knowledge and practice of patients with type 2 diabetes concerning dietary intake and blood glucose control in an outlying city of China. Public Health Nutr.

[37] Shamsudin J, Harith S, Razak SA, Zainal NA. A tailored dietary counselling via Diet Management Tool (DMT) helps dietitian improves short term glycaemic control among type 2 diabetes patients. Health Sci J. 2016;10(4):1–8.

[38] Neelapaichit N, Kaveevivitchai C, Piaseu N (2017). Preliminary study of effects of diet control program using food exchange on knowledge, food consumption behaviors, and glycemic control among persons with type 2 diabetes. bkkmedj.

[39] Al-Sinani M, Min Y, Ghebremeskel K, Qazaq HS. Effectiveness of and adherence to dietary and lifestyle counselling: effect on metabolic control in type 2 diabetic Omani patients. Sultan Qaboos Univ Med J. 2010;10(3):341–9. PMC3074742 21509254

[40] Ozcariz SG, de Bernardo OC, Cembranel F, Peres MA, González-Chica DA (2015). Dietary practices among individuals with diabetes and hypertension are similar to those of healthy people: a population-based study. BMC Public Health.

[41] Tol A, Mohebbi B, Sadeghi R, Maheri AB, Eshraghian MR (2014). Determinants of health-promoting behaviors among type 2 diabetic patients: voice of Iran. OJEMD..

[42] Agborsangaya CB, Gee ME, Johnson ST, Dunbar P, Langlois M-F, Leiter LA, Pelletier C, Johnson JA (2013). Determinants of lifestyle behavior in type 2 diabetes: results of the 2011 cross-sectional survey on living with chronic diseases in Canada. BMC Public Health.

[43] Shamsi N, Shehab Z, AlNahash Z, AlMuhanadi S, Micgp FA. Factors influencing dietary practice among type 2 diabetic patients in Bahrain. Bahrain Med Bull. 2013;35(3):1–13.

[44] Vizcarra M, Palomino AM, Iglesias L, Valencia A, Gálvez Espinoza P, Schwingel A (2019). Weight matters—factors influencing eating behaviors of vulnerable women. Nutrients..

[45] Li J, Powdthavee N (2015). Does more education lead to better health habits? Evidence from the school reforms in Australia. Soc Sci Med.

[46] Berhe KK, Demissie A, Kahsay AB, Gebru HB. Diabetes self care practices and associated factors among type 2 diabetic patients IN Tikur Anbessa Specialized Hospital, Addis ababa, Ethiopia- a cross sectional study. IJPSR. 2012;3(11):4219–29.

[47] Muñoz-Pareja M, León-Muñoz LM, Guallar-Castillón P, Graciani A, López-García E, Banegas JR (2012). The diet of diabetic patients in Spain in 2008–2010: accordance with the main dietary recommendations—a cross-sectional study. Sun Q, editor. PLoS ONE.

