# Supplementary material for: Eating behavior among persons with type 2 diabetes mellitus in North Ethiopia: a cross-sectional study
Source: BMC Endocr Disord. 2021 May 17;21:99. doi: 10.1186/s12902-021-00750-5 (PMC8127210; doi:10.1186/s12902-021-00750-5)
Supplement: Supplementary file 1 — Additional file 1. Demographic background and nutrition information of participants. [file 12902_2021_750_MOESM1_ESM.docx]

1. **Demographic background and nutrition information of participants**

| **No** | **Question** | **Response** | | **Go to/Skip** |
| --- | --- | --- | --- | --- |
| 101 | Age |  | _____ |  |
| 102 | Sex | Male  Female |  |  |
| 103 | Nationality |  | ________ |  |
| 104 | Ethnicity | Tigrian  Amhara  Oromo  Afar  Other (specify) |  |  |
| 105 | Religion | Orthodox  Muslim  Catholic  Protestant  Others (specify) |  |  |
| 106 | Marital status | Single  Married  Widowed  Divorced |  |  |
| 107 | Educational status | Illiterate  Able to read and write  Primary school  Secondary school  College graduate or above |  |  |
| 108 | Did you get any nutritional education or advice regarding dietary control of diabetes so far? | Yes  NO | 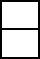 | If the response is NO, Skip Q109 |
| 109 | Who gave it to you?  (More than one response is allowed) | Doctor  Nurse  Nutritionist  Other team of health professionals as a community service | 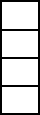 |  |
