# Supplementary material for: Eating behavior among persons with type 2 diabetes mellitus in North Ethiopia: a cross-sectional study
Source: BMC Endocr Disord. 2021 May 17;21:99. doi: 10.1186/s12902-021-00750-5 (PMC8127210; doi:10.1186/s12902-021-00750-5)
Supplement: Supplementary file 2 — Additional file 2. Eating behavior questionnaire (food selection, meal planning and calorie needs recognition). [file 12902_2021_750_MOESM2_ESM.docx]

**3. Eating behavior questionnaire (food selection, meal planning and calorie needs recognition)**

| **Eating behavior dimensions and items** | | **Response** | | | |
| --- | --- | --- | --- | --- | --- |
|  |  | Strongly disagree | Disagree | Agree | Strongly agree |
| **Food selection dimension** | |  |  |  |  |
| 301 | You choose foods that contain low to medium glycemic index in your diet |  |  |  |  |
| 302 | You or the person who cooks for you rarely uses saturated fats for cooking |  |  |  |  |
| 303 | You eat fruits and vegetables every day |  |  |  |  |
| 304 | You avoid salty diet |  |  |  |  |
| 305 | You avoid or take alcohol in moderation |  |  |  |  |
|  | **Meal planning dimension** |  |  |  |  |
| 306 | You understand and able to arrange your right meal plan |  |  |  |  |
| 307 | You understand and able to use plate methods in arranging your meal plan within a day as follows:   - 1^1^/_2_ plate of vegetables - A plate of meat or meat substitutes - A plate of starch - 3 glasses of milk/fruit or 1 fruit - Oils or butter sparingly |  |  |  |  |
| 308 | You understand and able to use food exchange list in arranging your meal plan |  |  |  |  |
| 309 | You eat 3 meals and 3 snacks a day |  |  |  |  |
| 310 | You eat meal in the same time every day |  |  |  |  |
| 311 | You eat a variety of foods in every meal daily that include the following  -Fleshy foods (fish, chicken or meat)  - Cereals (Teff, wheat, barley)  - Low fat  - Vegetables (Spinach, Lettuce, Cabbage)  - Fruits (Orange, Mango, Papaya)  - Pulses (Beans, Peas, lentils) |  |  |  |  |
|  | **Calorie needs recognition dimension** |  |  |  |  |
| 312 | You know and maintain the calorie proportions you should take in each meal from:  - Carbohydrate  - Protein  -Fat |  |  |  |  |
| 313 | You weight and measure calorie of food in each meal using cups, grams or serving sizes |  |  |  |  |
| 314 | You consume same amount of food every day |  |  |  |  |
